# A randomised trial of single-dose radiotherapy to prevent procedure tract metastasis by malignant mesothelioma

**DOI:** 10.1038/sj.bjc.6601957

**Published:** 2004-06-15

**Authors:** S Bydder, M Phillips, D J Joseph, F Cameron, N A Spry, Y DeMelker, A W Musk

**Affiliations:** 1 Department of Radiation Oncology, Sir Charles Gairdner Hospital, Perth, Australia; 2 Department of Respiratory Medicine, Sir Charles Gairdner Hospital, Perth, Australia; 3 School of Population Health, University of Western Australia, Perth, Australia

**Keywords:** mesothelioma, radiotherapy, neoplasm seeding

## Abstract

A single 9-MeV electron treatment, following invasive thoracic procedures in patients with malignant pleural mesothelioma, was examined. In all, 58 sites were randomised to prophylactic radiotherapy or not. There was no statistically significant difference in tract metastasis. A single 10-Gy treatment with 9-MeV electrons appears ineffective.

In recent years, the incidence of malignant pleural mesothelioma (MPM) has been increasing rapidly (Peto *et al*, 1999). Chest wall seeding following invasive procedures is a problem, and has been reported to occur in 19% of patients following thoracoscopy (Boutin *et al*, 1995). These subcutaneous masses are often symptomatic and refractory to radiotherapy (RT) (Davis *et al*, 1994). Two nonrandomised series and one randomised controlled trial have demonstrated that a prophylactic three-fraction course of RT reduces the procedure tract metastasis rate to 0% (Boutin and Rey, 1993; Boutin *et al*, 1995; Low *et al*, 1995). Recent reviews and guidelines recommended prophylactic radiotherapy following thoracic procedures (BTSSCC, 2001; Parker and Neville, 2003). We undertook a randomised trial to test a more convenient single radiation treatment.

## METHODS

### Patients

The eligibility criteria included: histological confirmation of MPM; age greater than 18 years; and, a clearly identifiable procedure site. Written informed consent was obtained from all trial patients. The study had received institutional ethics committee approval.

### Prophylactic radiation treatment

Patients were randomised after stratification by procedure type, to receive either a single dose of electron beam chest wall radiotherapy or no prophylactic therapy. A dose of 10 Gy in a single fraction was delivered to the chest wall, using 9-MeV electrons. The dose was prescribed at 100%. No bolus was used. Radiotherapy was given within 15 days of thoracic procedures.

### Data collection and follow-up

In all cases, the site of the procedure was recorded, tattooed and photographed, to allow accurate subsequent assessment. Physicians assessed patients clinically for masses in the region of the procedure, at 3 and 6 months, then 6-monthly until death. Acute and late radiation toxicities were assessed and graded according to the RTOG/EORTC criteria (Cox *et al*, 1995). Patients were assessed for early radiation toxicity at 1 week by the treating radiation oncologists, and subsequently for late radiation effects by the respiratory physicians.

### Statistical methods

The primary outcome measure of the trial was procedure tract metastasis. No effect on overall survival of the patients was expected. The trial was designed with 80% power to detect a 20% reduction in tract metastasis with prophylactic chest wall irradiation (i.e. from an expected 20 to 0%) at the 5% one-sided alpha significance level. The estimated number of tract metastases following prophylactic RT was based on three published studies, all reporting no failures. The calculated minimum sample size of 54 sites was increased to 58 to allow for patients who might be lost to follow-up. Tract metastasis-free survival was defined from the time of randomisation to the development of a clinically apparent metastasis in the immediate region of the procedure site or to the last follow-up information. Overall survival was defined as the interval from the date of randomisation to the date of death or the last follow-up information. The crude incidence of tract metastasis in the two trial arms was compared using Fisher's exact test (this was one-tailed, as radiotherapy will not increase the rate of tract metastasis). Tract metastasis-free survival was compared by log rank testing. The crude tract failure rates for the three-procedure strata were compared using a two-sided Fisher's exact test. All analyses were performed on an intent-to-treat basis, and were performed with the use of SPSS (Chicago, IL) statistical software.

## RESULTS

Between December 1997 and July 2003, 58 procedure sites were registered into the trial, from 43 patients; 28 sites were randomised to the prophylactic chest wall RT arm and 30 sites to the control arm. In all, 10 patients had two, and one patient three, sites separately randomised. The clinical characteristics of the 58 sites are summarised in Table 1

**Table 1:** The characteristics of sites registered on study

	**RT arm (*n*=28)**	**Control (*n*=30)**	**Overall**
	**Number (%)**	**Number (%)**	
*Age (years)*			
Mean	70.0	70.0	
Range	53.7–85.1	51.0–86.3	
			
*Sex*			
Male	26 (93%)	29 (97%)	55
Female	2 (7%)	1 (3%)	3
			
*Procedure*			
Thoracic drain/thoracoscopy	11 (39%)	11 (37%)	22
FNA	13 (46%)	14 (47%)	27
Abrams needle	4 (14%)	5 (17%)	9
			
*RT given*			
	28 (100%)	1 (3%)	

. Overall, 22 sites had undergone thoracic drainage or thoracoscopy, nine sites Abrams needle and 27 sites fine needle aspiration (FNA) prior to randomisation. One patient in the control arm was given prophylactic radiotherapy at his request. The radiotherapy fields used ranged from 4- to 8-cm diameter circles.

Both early and late toxicity following radiotherapy were mild with no RTOG/EORTC grade 2, 3 or 4 reactions noted. The overall median survival from randomisation was 8.7 months, with a 1-year survival of 35%. Only seven patients remain alive.

There was no statistically significant difference in tract metastasis between the two arms of the trial, with three (10%) metastases in the control arm and two (7%) in the prophylactic RT arm (*P*=0.53). The freedom from tract metastasis survival for the two arms was not significantly different on log rank testing (*P*=0.82). The crude rates of tract metastases overall were 22% for Abrams needles, 9% for thoracic drains and 4% for FNA, and these were not statistically significantly different (*P*=0.23).

## DISCUSSION

We performed a randomised controlled trial examining the effect of a single dose of prophylactic chest wall radiotherapy following invasive procedures in MPM. There was no statistically significant difference in procedure tract metastases. Our study was relatively large with precise follow-up, and included patients who had undergone a range of procedures.

Tract metastases in the control arm of the trial were low. The median rate in the literature following thoracoscopies is 19%. Similar tract metastasis rates are reported for Abrams and other large needles (Metintas *et al*, 1995; Roussel and Nouvet, 1995). Pleurectomy and extra-pleural pneumonectomy have high rates of chest wall failure without prophylactic RT (Rusch *et al*, 2001). There is little information on tract metastasis following fine needle aspiration, but in our study this procedure carried the lowest risk of malignant seeding (although this was not statistically significantly). The risk of seeding appears to be related to procedure and technique.

A dose of 10 Gy delivered in a single fraction using 9-MeV electrons does not appear to be effective in preventing procedure tract metastasis. The relative effective dose for different radiation schedules can be estimated using the linear-quadratic model (Muller-Runkel and Vijayakumar, 1991). For cancers, 10 Gy in a single fraction is equivalent to delivering 12 Gy in six 2-Gy fractions. For comparison, 21 Gy in three fractions is equivalent to approximately 42 Gy in 21, 2-Gy fractions. That is, the dose we used was equivalent to approximately 40% of the reported three-fraction schedules. This lower dose could be expected to be less effective, but the actual difference in outcome depends on steepness of the tumour control probability curve. Our study suggests a clinically important dose–response over this dose range.

Another possible explanation for the lack of effect is that 9 MeV electrons might be inadequately penetrating. The French nonrandomised series and subsequent randomised trial used higher energy 12–15 MeV electrons (Boutin and Rey, 1993; Boutin *et al*, 1995), while the British study employed 140- or 250-kV photons (Low *et al*, 1995). These alternative radiations give a higher dose beyond approximately 3 cm depth in tissue. However, 9 (or less) MeV electrons successfully deliver post-mastectomy chest wall radiotherapy (e.g. Morgan *et al*, 2002; Feigenberg *et al*, 2003), and this seems a less likely explanation.

We did not demonstrate a benefit from a single dose of prophylactic chest wall irradiation. We continue to recommend prophylactic treatment to be used following high-risk procedures, delivering 21 Gy in three fractions.
